# NSAID-Associated Small Intestinal Injury: An Overview From Animal Model Development to Pathogenesis, Treatment, and Prevention

**DOI:** 10.3389/fphar.2022.818877

**Published:** 2022-02-09

**Authors:** Mingyu Zhang, Feng Xia, Suhong Xia, Wangdong Zhou, Yu Zhang, Xu Han, Kai Zhao, Lina Feng, Ruonan Dong, Dean Tian, Yan Yu, Jiazhi Liao

**Affiliations:** ^1^ Department of Gastroenterology, Tongji Hospital of Tongji Medical College, Huazhong University of Science and Technology, Wuhan, China; ^2^ Institute of Liver and Gastrointestinal Diseases, Tongji Hospital of Tongji Medical College, Huazhong University of Science and Technology, Wuhan, China; ^3^ Department of Hepatic Surgery Center, Tongji Hospital of Tongji Medical College, Huazhong University of Science and Technology, Wuhan, China

**Keywords:** NSAIDs, enteropathy, model, pathogenesis, toxicology, emerging therapy

## Abstract

With the wide application of non-steroidal anti-inflammatory drugs (NSAIDs), their gastrointestinal side effects are an urgent health burden. There are currently sound preventive measures for upper gastrointestinal injury, however, there is a lack of effective defense against lower gastrointestinal damage. According to a large number of previous animal experiments, a variety of NSAIDs have been demonstrated to induce small intestinal mucosal injury *in vivo*. This article reviews the descriptive data on the administration dose, administration method, mucosal injury site, and morphological characteristics of inflammatory sites of various NSAIDs. The cells, cytokines, receptors and ligands, pathways, enzyme inhibition, bacteria, enterohepatic circulation, oxidative stress, and other potential pathogenic factors involved in NSAID-associated enteropathy are also reviewed. We point out the limitations of drug modeling at this stage and are also pleased to discover the application prospects of chemically modified NSAIDs, dietary therapy, and many natural products against intestinal mucosal injury.

## 1 Introduction

Non-steroidal anti-inflammatory drugs (NSAIDs) are among the most widely used drugs worldwide (Scarpignato and Hunt, 2010). Their most common side effects are gastric and intestinal mucosal damage. In the past, attention was focused only on preventing gastric complications. In recent years, the occurrence of NSAID-associated enteropathy has received increasing attention, especially after the advent of enteric-coated preparations of NSAIDs. Data have reported that severe damage to the small intestine accounts for one-third of NSAID complications (Scarpignato and Hunt, 2010). NSAID-associated enteropathy involves the jejunum and ileum, manifested by mucosal erythema, erosion, ulceration, annular constriction ring formation, or even hemorrhage and obstruction (Endo et al., 2015; Smecuol et al., 2009; Sostres et al., 2013). With the aging population in China, the use of NSAIDs will continue to increase, and conquering the side effects of small intestinal injury has gradually become a clinical problem. Treatment and preventive measures need to be taken as soon as possible.

A convenient way to study the pathogenesis and complexity of NSAID-associated enteropathy in humans is to establish animal models. Although disease models established using animals cannot wholly represent human disease, we can study the typical pathological features they present with. Animal models of NSAID-associated enteropathy are essential for better understanding the histopathology of the small intestine and the causative factors at different stages. In addition, animal models play a significant role in exploring effective therapeutic and preventive measures and analyzing therapeutic modalities’ possible mechanisms of action. Researchers have successfully used a wide variety of NSAIDs to establish disease models in rodents, including indomethacin (Kawashima et al., 2020; Kuzumoto et al., 2021; Tawfik et al., 2016; Yamada et al., 1993), diclofenac (Beck et al., 1990; Chen et al., 2021; Xu et al., 2021), aspirin (Bouzenna et al., 2019; Brodie et al., 1970a; Chi et al., 2021), flurbiprofen (Campanella and Jamali, 2009), naproxen (Carvalho et al., 2015; Nicolau et al., 2017), loxoprofen (Hayashi et al., 2013; Satoh and Urushidani, 2016), ibuprofen (Beck et al., 1990; Lu et al., 2018), diflunisal (Niu et al., 2014), BFMeT (Hagiwara et al., 2004; Uejima et al., 1996), paracetamol (Chopyk et al., 2019; Niu et al., 2020), Ketoprofen (Cheng et al., 2014; Saitta et al., 2014). Many experimental articles have verified the reproducibility and stability of various modeling modalities. This review discusses various models of NSAID-induced small intestinal inflammation to profoundly investigate the pathogenesis of NSAID-associated enteropathy and seek meaningful therapeutic strategies.

## 2 Models of NSAIDS Induced Small Intestinal Inflammation

In this section, we mention a variety of NSAIDs that can cause damage to the small intestinal mucosa. We review the dosing regimens and measured variables in these papers in Table 1.

**TABLE 1:** NSAIDs induced models of intestinal inflammation.

NSAID	Method	Animal	Injury manifestations	References
Indomethacin	10 mg/kg p.o.	Male C57BL/6 mice	Macroscopic and microscopic damage	Kuzumoto et al. (2021)
	30 mg/kg s.c.	Male BALB/cAJcl mice	Weight loss; food intake decrease; fecal volume decrease; Changes in stool properties; histologic heterogeneity of villi and crypt; cell infiltration increase	Kawashima et al. (2020)
	7.5 mg/kg once daily for 2 days s.c.	Male SD rats	A more severe chronic inflammatory response including ulcers, adhesion, bleeding, bowel dilatation and thickening	Yamada et al. (1993)
	7.5 mg/kg once daily for 2 days s.c.	Male albinorats	Weight loss; small intestinal length decrease; adhesions; hyperemia; edema; ulcers; hemorrhage; changes in villus structure	Tawfik et al. (2016)
Diclofenac	15 and 30 mg/kg p.o./i.v.	Male SD rats	Macroscopically visible damage	Beck et al. (1990)
	7.5 mg/kg twice a day for 5 days p.o.	Male SD rats	Mucosal congestion; edema; erosion; ulceration; villous atrophy; irregular glandular arrangement; inflammatory cell infiltration	Chen et al. (2021)
	10 mg/kg once daily for 4 days p.o.	Male SD rats	Bloody stools; intestinal adhesion; intestinal perforation; absence of villi; disordered gland; erosion; ulcer; hemorrhage	Xu et al. (2021)
Aspirin	300 and 1,000 mg/kg p.o.	Male ddy mice	Reddish areas and mild erosions	Satoh and Urushidani (2016)
	10.41 mg/kg/d for 14 days p.o.	Male SD rats	Scattered punctate erosion; sheet erosion; adhesion; villi and glandular epithelium atrophy; monocytes infiltration	Chi et al. (2021)
	50–200 mg/body injected into duodenum	Male SD rats	Severe bleeding and ulcers mainly in the jejunum	Nonoyama et al. (2010)
Flurbiprofen	2.5 mg/(kg 12 h) i.p./p.o.	Male SD rats	Gut permeability increase; “adaptive” effect	Campanella and Jamali (2009)
Naproxen	80 mg/kg, p.o. Twice daily for 2 days	Male Wistar rats	Significant villi shortening; increased crypt depth	Carvalho et al. (2015), Nicolau et al. (2017)
Loxoprofen	100 and 300 mg/kg s.c.	Male ddy mice	Marked lesions mainly in the lower small bowel; decreased intestinal length	Satoh and Urushidani (2016)
	60 mg/kg p.o.	Male SD rats	Multiple hemorrhagic lesions mainly in the ileum; lesions reaching the muscularis mucosae	Hayashi et al. (2013)
Ibuprofen	100 mg/kg p.o.	Male SD rats	Macroscopically visible damage	Beck et al. (1990)
	200 mg/kg p.o.	Male C57BL/6 mice	Haemorrhagic damage and massive loss of villi	Lu et al. (2018)
Diflunisal	1 mM Incubation	PCIS from male Wistar rats	Severe loss of epithelial cell	Niu et al. (2014)
BFMeT	250 mg/kg p.o.	Male Wistar rats	Ileal ulcers formation	Hagiwara et al. (2004)
	500 mg/kg or higher p.o.	Male Wistar rats	Ileal ulcers formation; perforated ulcers; conglutinated small intestines; bulging of the intestines; shrunken cecum; ascites	Uejima et al. (1996)
Paracetamol	500 mg/kg i.p.	Male C57BL6/J mice	Gut permeability increase; sparse apoptotic bodies remained within ileum sections	Chopyk et al. (2019)
	300 mg/kg i.p.	Male C57BL/6 J mice	Gut permeability increase	Niu et al. (2020)
Ketoprofen	100 mg/kg i.p.	Male C57BL/6 J mice	Mild jejunal ulceration; 5/10 mice were non-responders	Saitta et al. (2014)
	50 mg/kg p.o.	Male SD rats	Ulcers, cloudy, swelled areas, necrosis, lymphocyte infiltration	Cheng et al. (2014)

Abbreviations: Oral administration (p.o.); Intraperitoneal injection(i.p.); Intravenous administration(i.v.); Subcutaneous injection(s.c.).

### 2.1 Indomethacin

As early as 1969, researchers accidentally discovered intestinal damage while studying the anti-inflammatory effect of indomethacin (Kent et al., 1969; Somogyi et al., 1969). SD rats received single doses of 1, 2, 4, and 8 mg indomethacin into the jugular vein. Numerous circular or elongated ulcers could be observed in the jejunum (Somogyi et al., 1969). Most rats died after indomethacin injection. Because it was a preliminary experiment, a reliable animal model of indomethacin-associated enteropathy needed to be established. Over time researchers have found the appropriate dose and the proper method of administration. Gavage and subcutaneous injection are common. 10 mg/kg body weight of indomethacin suspended in a 0.5% carboxymethylcellulose solution was orally administered to C57BL/6 mice to induce small intestinal damage (Kuzumoto et al., 2021). Indomethacin was dissolved in 0.03 M NaOH, neutralized with 0.05 M HCl, and suspended in saline. It was administered by subcutaneous injection once (30 mg/kg) in BALB/cAJcl mice (Kawashima et al., 2020). Subcutaneous injection of indomethacin twice daily produces more extensive and chronic inflammation (Yamada et al., 1993). The clinical features of this model include weight loss, decreased food intake and decreased fecal volume. Pathological scoring is generally performed in the lower half of the small intestine. Histopathological analysis typically reveals increased histologic heterogeneity of the jejunal villi and crypts, and significant infiltration of granulocytes and mononuclear immune cells. Histopathology, by hematoxylin and eosin staining, was scored based on several parameters: the destruction of epithelium and glands, dilation of glandular crypts, depletion and loss of goblet cells, inflammatory cellular infiltration, edema, hemorrhagic mucosa, and crypt abscesses (Tawfik et al., 2016). Indomethacin chemically complexed with phosphatidylcholine prevented acute gastrointestinal damage (Dial et al., 2015).

### 2.2 Diclofenac

In 1990, some researchers explored gastrointestinal ulcers caused by anti-inflammatory drugs. They found that oral administration of diclofenac (15 or 30 mg/kg body weight) or intravenous injection of diclofenac (30 mg/kg body weight) could cause significant damage in the small intestine. However, the ulcerogenic effect of diclofenac was not as strong as that of indomethacin (Beck et al., 1990). Now, diclofenac is one of the most prescribed drugs worldwide, and it is also one of the commonly used modeling drugs for NSAID-related enteropathy. In a diclofenac-induced rat model, intestinal tissue mucosal congestion, edema, erosion, and even ulceration could be observed (Chen et al., 2021). The intestinal injury was evaluated in ulceration, adhesion to the surrounding tissue, and diarrhea using the Reuter BK scoring method. Microscopic histological sections of the intestine showed shortened villi, disorganized glands, and extensive infiltration of inflammatory cells in the small intestine, which was assessed using Chiu’s 6-grade scoring method (Xu et al., 2021).

### 2.3 Aspirin

Aspirin is the most widely prescribed nonsteroidal anti-inflammatory drug (NSAID) worldwide. As early as 1970, D A Brodie and his colleagues used a blue dye so that small intestinal damage in rats that were orally administered aspirin could be observed grossly. At that time, aspirin-related enteropathy had not received much attention (Brodie et al., 1970b). With the development of capsule endoscopy, recent studies suggest that long-term low-dose aspirin use is associated with lower digestive tract injury. It is an essential factor in developing small intestinal ulcers, bleeding, and stenosis (Endo et al., 2014; Watanabe et al., 2008a). H. Satoh et al. treated cats with aspirin by gavage for 1 week and found lesions in the small intestine, but cats are not commonly used experimental animals. They are much more complex to treat than mice, making this model unsuitable for a wide range of applications (Satoh et al., 2013). Then they tried to give high-dose aspirin to mice and found that the antrum was more prone to develop a severe injury, but small intestinal lesions were mild (Satoh and Urushidani, 2016). Oral administration of aspirin (10.41 mg/kg/d) for 14 days only induced intestinal scattered erosions in SD rats (Chi et al., 2021). Aspirin does not appear to cause severe damage to the intestinal mucosa of experimental animals. Aspirin is rapidly absorbed in the stomach and duodenum, and it lacks enterohepatic circulation, so its concentration in the distal small intestinal lumen is low (Somasundaram et al., 1997). It has been reported that bleeding and ulcers could be observed in the jejunum after a single aspirin injection directly into the duodenum of rats (Nonoyama et al., 2010). However, this model is cumbersome to construct. It ignores the interaction of drugs with the stomach and does not properly mimic the process oral medications pass through in humans. On establishing animal models of aspirin-associated enteropathy, we should continue to explore better ways.

### 2.4 Flurbiprofen

Flurbiprofen increased intestinal permeability in rats after intraperitoneal or oral administration, confirming the systemic and local effects of the drug. Interestingly, investigators have found that this drug has an “adaptive” effect, that is, with continuous application of the same dose, there is a gradual decrease in intestinal permeability (Campanella and Jamali, 2009). Its ulcerogenic potency was significantly reduced after flurbiprofen was modified into an amino-alcohol ester derivative. Chemical modification of drugs effectively reduces their gastrointestinal toxicity (Halen et al., 2006).

### 2.5 Naproxen

Oral administration of naproxen (80 mg/kg) to rats for 2 days resulted in medial intestinal injury, shortening of villi, and deepening of crypts (Carvalho et al., 2015; Nicolau et al., 2017). Naproxen modified by H2S had significantly reduced gastrointestinal toxicity (Wallace et al., 2015; Wallace et al., 2010).

### 2.6 Loxoprofen

Subcutaneous injection of indomethacin (100 or 300 mg/kg) could produce small intestinal injuries in unfasted mice. However, approximately ten times the dose of loxoprofen was required to produce the same ulcerogenic effect as indomethacin with loxoprofen (Satoh and Urushidani, 2016). Multiple hemorrhagic lesions could be observed macroscopically. The lesions induced by loxoprofen were mainly located in the deep layer of the ileal mucosa (Hayashi et al., 2013).

### 2.7 Ibuprofen

Oral administration of 100 mg/kg ibuprofen to SD rats produced grossly visible small intestinal mucosal damage (Beck et al., 1990). A combination of high-dose dexamethasone and ibuprofen exacerbates small intestinal mucosal injury (Lu et al., 2018). New drugs of ibuprofen, which chemically bind to phosphatidylcholine, had a higher gastrointestinal safety while their therapeutic effect was unabated (Lanza et al., 2008).

### 2.8 Diflunisal

The researchers used precision-cut intestinal slices (PCIS) derived from Wistar rats to assess NSAID toxicity. Typical tissue damage could be observed in diflunisal-induced precision-cut intestinal slices, indicating the high intestinal toxicity of diflunisal (Niu et al., 2014). However, no intestinal injury had been seen in the *in vivo* models (Beck et al., 1990). After all, drugs undergo absorption, transformation, and catabolism in the body. There are still considerable differences between drug effects *in vivo* and *in vitro*. To some extent, it shows the limitations of the *ex vivo* model.

### 2.9 BFMeT

It has been documented that 5-bromo-2-(4-fluorophenyl)-3-(4-methylsulfonylphenyl) thiophene (BFMeT, a non-acidic NSAID) was used to induce ileal ulcers in rats successfully (Hagiwara et al., 2004; Uejima et al., 1996). The clinical features of this model include perforated ulcers, conglutinated small intestines, bulging of the intestines in the area of ulceration, a shrunken cecum, and ascites.

### 2.10 Paracetamol

Paracetamol leads to intestinal damage by inducing a process of apoptotic cell death of LGR5+ stem cells in the intestinal crypts, and the intestinal barrier function of paracetamol-treated mice is affected for a long time (Chopyk et al., 2019). Paracetamol administration leads to increased intestinal permeability as well as CCL7 upregulation, which promotes drug-induced liver injury (Niu et al., 2020).

### 2.11 Ketoprofen

Although ketoprofen and indomethacin are both carboxylic acid-containing NSAIDs, ketoprofen is significantly milder in causing small intestinal mucosal injury (Saitta et al., 2014). Administration of ketoprofen 50 mg/kg/day to SD rats could cause oxidative gastrointestinal mucosal injury. Local villus loss, edema, necrosis, and lymphocytic infiltration could be observed microscopically (Cheng et al., 2014).

## 3 Factors Involved in the Pathogenesis of the NSAID-Associated Enteropathy

This section focuses on displaying the pathogenetic factors involved in the above NSAID-associated enteropathy one by one.

### 3.1 COX Inhibition

Cyclooxygenase (COX) is a crucial enzyme catalyzing the formation of prostaglandins. At present, it has been found that COX has two isoenzymes. COX-1 is a constitutive type, mainly present in blood vessels, stomach, kidney, and other tissues, involved in the regulation of vascular relaxation, platelet aggregation, gastric mucosal blood flow, gastric mucus secretion, and renal function. COX-2 is an inducible type. Various chemical, physical, and biological factors activate phospholipase A2 to hydrolyze cell membrane phospholipids to generate arachidonic acid, which COX-2 catalyzes to generate prostaglandins (Kargman et al., 1996; Vane et al., 1998).

COX inhibition alone was insufficient to induce small intestinal injury in rats (Menozzi et al., 2006). The pathogenesis of indomethacin-induced NSAID enteropathy seems to be less dependent on COX inhibition. Anyway, COX inhibition is accompanied by a reduction in prostaglandins, which may alter local blood flow and therefore be an essential co-factor driving the progression of inflammation to ulceration (S et al., 2014). The inhibition of COX could transform low-grade inflammation into ulcers (Somasundaram et al., 2000). The inhibition of COX does appear to contribute to the development of ulcerations in the small intestine (Beck et al., 1990). In addition, in acquired immunodeficiency syndrome (AIDS) patients treated with protease inhibitors plus NSAID, the prostaglandin-depleted environment may allow ritonavir to aggravate intestinal damage (Renga et al., 2014). Indomethacin-induced intestinal lesions were significantly prevented by prior intravenous administration of PGE analogs. The protective effect of exogenous PGE2 is receptor-specific. It prevents the intestinal ulcerogenic response to indomethacin through different EP receptor subtypes. EP3/EP4 receptors mediate the protection in the intestine (Kunikata et al., 2001; Kunikata et al., 2002).

### 3.2 Uncoupling of Mitochondrial Oxidative Phosphorylation and Oxidative Stress

NSAIDs induced mitochondrial alterations in a dose-dependent manner, and the mechanism of NSAID-induced intestinal injury involved uncoupling of oxidative phosphorylation or inhibition of electron transport (Somasundaram et al., 1997; Somasundaram et al., 2000). Diazoxide exerted a protective effect against mitochondrial damage by opening mitoK_ATP_ channels, which prevented phosphorylation uncoupling (Menozzi et al., 2011).

Oxidative stress is one of the mechanisms of NSAID-induced small intestinal injury. Rats administered with indomethacin or diclofenac had a marked increase in intestinal oxidative stress (Fornai et al., 2014). Diclofenac could uncouple mitochondrial oxidative phosphorylation and elevate superoxide anion in a dose-dependent manner (Bhatt et al., 2018). Exposure of intestinal epithelial cells to indomethacin leads to a significant reactive oxygen species production, which can be inhibited by antioxidant pretreatment (Omatsu et al., 2009). Melatonin effectively scavenged superoxide anions and improved enterocyte redox status to prevent NSAID-induced intestinal mucosal disruption (Sanchez et al., 2020). In addition, Catechins belong to the polyphenolic compounds in phytochemical constituents, which are natural antioxidants in foods. Catechins were able to alleviate ketoprofen-induced oxidative damage in the intestinal mucosa of SD rats (Cheng et al., 2013). Essential oil from Citrus limon is obtained by extraction or distillation of volatile plant molecules, pretreatment with essential oil from Citrus limon provided significant protection against aspirin-induced intestinal injury in rats, and the protective effect could be attributed to its antioxidant activity (Bouzenna et al., 2019). Epiisopiloturine hydrochloride is a natural product isolated from Pilocarpus microphyllus leaves. It effectively scavenged free radicals, reduced lipid peroxidation, and played an ameliorative role in naproxen-induced gastrointestinal damage (Nicolau et al., 2017). Cashew gum is extracted from the trunk of the Anacardium occidentale L. tree, and it showed the inhibition of glutathione level depletion and the blockade of naproxen-induced intestinal oxidative stress (Carvalho et al., 2015). These natural products with antioxidant activity provide a novel therapeutic strategy for preventing NSAID-associated enteropathy in patients. Besides, the synthetic dexibuprofen-antioxidant conjugates had fewer gastrointestinal side effects while being pharmacologically active as the parent drug (Ashraf et al., 2016). It is reported that polaprezinc, an antioxidant, effectively reduced the number of reddened lesions and ulcers caused by low-dose aspirin (Watari et al., 2013). The prevention and treatment of NSAID-related enteropathy can be further explored in the antioxidant field.

### 3.3 Enterohepatic Circulation

#### 3.3.1 Hepatobiliary Excretion of NSAIDs

NSAID-induced intestinal epithelial damage is due to direct effects after oral administration, recurrent local effects are due to enterohepatic recirculation of the drug, and systemic effects occur after absorption (Lanas and Sopena, 2009). Enterohepatic circulation refers to the phenomenon that drugs excreted into the intestine through bile are reabsorbed in the intestine and return to the liver through the portal vein (Gao et al., 2014). Bile duct ligation attenuated high dose indomethacin-induced intestinal perforation and death (Brodie et al., 1970a). Somasundaram et al. observed alterations in mitochondrial integrity of intestinal epithelial cells in indomethacin-treated rats, and bile duct ligation could prevent this early alteration (Somasundaram et al., 1997). In rats, four different NSAIDs were used to reveal a close relationship between the degree of enteropathy and the amount of drug secreted in the bile (Beck et al., 1990). All these experiments show that the enterohepatic circulation of drugs is one of the pathogenetic factors. Local effects of drugs are more important than systemic effects in the ulcerogenic effects of NSAIDs.

Intravenous administration of either diclofenac or ibuprofen could increase the bile flow to some extent (Beck et al., 1990). The combination of NSAIDs with bile seemed to produce more significant cytotoxicity toward the small intestine than either component alone (Yamada et al., 1993; Zhou et al., 2010). Bile acids had a permissive effect on the ulcerogenic effect of indomethacin. Microorganism-induced conversion of primary to secondary bile acids may be an important driving force (Weissenborn et al., 1985). The relatively toxic secondary bile acids may be essential in NSAID-induced distal intestinal injury (Robert and Asano, 1977). However, in recent studies, ibuprofen or indomethacin did not increase the proportion of cytotoxic secondary bile acids in the distal small intestine. The proportion of secondary bile acids and the hydrophobic index of the small intestinal contents do not seem to play an essential role in NSAID-associated enteropathy (Lazar et al., 2021; Lu et al., 2018). The contradiction of these conclusions is blamed on the fact that bile from different sites (biliary tract and small intestine) was collected for bile acid analysis. More experiments are needed to investigate the causal relationship between bile acid composition and intestinal mucosal injury. Perhaps factors other than altered bile acid ratio are involved in the increased NSAID-mediated bile toxicity.

Unlike other NSAIDs, aspirin is not excreted in the bile (Reuter et al., 1997). In a randomized controlled study, low-dose aspirin could cause significant small intestinal mucosal injury (Watari et al., 2013). Perhaps the inhibition of COX and uncoupling of mitochondrial oxidative phosphorylation play a dominant role, and the enterohepatic circulation of NSAIDs is not crucial in the pathogenesis of NSAID enteropathy (Somasundaram et al., 2000). Although aspirin was not excreted unchanged, excretion of its major metabolite, salicylic acid, could be detected in the bile (Beck et al., 1990). Perhaps its metabolite salicylic acid plays an ulcerogenic role, or aspirin causes damage by some mechanisms other than enterohepatic circulation.

Inhibition of bacterial β-d-glucuronidase activity *in vivo* using Inh-1 or ciprofloxacin has been shown to prevent the enterohepatic circulation of NSAIDs and reduce intestinal damage caused by these drugs (Kim et al., 2018; LoGuidice et al., 2012; Saitta et al., 2014; Zhong et al., 2016). The excretion of the drug in the bile was significantly reduced in rats treated with NO-diclofenac mixture compared to the rats treated with diclofenac only (Reuter et al., 1997). We can find more preventive measures for NSAID enteropathy from the perspective of inhibiting enterohepatic circulation processes.

#### 3.3.2 Bile Acid Receptors

There are many bile acid receptors involved in the regulation of NSAID-associated enteropathy:

Farnesoid X receptor (FXR) is a bile acid sensor. FXR gene ablated mice were more prone to develop severe intestinal injury in response to naproxen. FXR activation by GW4064 protected against small intestinal damage caused by naproxen (Fiorucci et al., 2011). The combination of dexamethasone and ibuprofen increased the concentration of a naturally occurring FXR antagonist in mice and significantly inhibited FXR signaling. Glucocorticoid receptor GR was involved in inhibiting FXR signaling in enterocytes co-administrated with dexamethasone and ibuprofen (Lu et al., 2018).

Key enzymes of the NSAID metabolic pathway, such as cytochrome P450 enzymes and UDP-glucuronosyltransferase, are regulated by the nuclear receptor progesterone X receptor (PXR). It has been reported that the use of PXR agonists does not aggravate the ibuprofen-induced intestinal injury, demonstrating that PXR signaling is not a causative factor in ibuprofen-induced enteropathy (Lu et al., 2018). PXR-deficient (Nr1i2 −/−) mice showed ultrastructural defects in the small intestine, increased intestinal permeability, upregulation of Toll-like receptor4, and higher sensitivity to indomethacin-induced intestinal injury compared with normal (Nr1i2 +/+) mice (Venkatesh et al., 2014). These findings reveal that PXR is an important modulator of intestinal homeostasis and plays a protective role.

The activation of bile acid receptor TGR5 on intestinal L cells indirectly up-regulates the expression of glucagon-like peptide GLP-2 and increases intestinal HCO3- secretion by amplifying the amino acid-taste receptor 1 family heterodimer T1Rs—calcium pathway, revealing its intestinal mucosal protective effect and predicting its intestinal ulcer treatment prospects (Inoue et al., 2012). It has been reported that TGR5 deletion significantly exacerbates naproxen-induced intestinal injury, and experimental results highlight the gastrointestinal mucosal protective effect of TGR5 (Cipriani et al., 2013). The utilization of TGR5-regulated pathways may represent a novel mechanism for treating NSAID-associated enteropathy.

In LPS activated RAW264.7 macrophages, the interaction of the ligand calcitroic acid/vitamin D with the vitamin D receptor (VDR) significantly reduced the expression of iNOS and IL-1β, reflecting the anti-inflammatory properties mediated by the VDR (Yu et al., 2021). A recent study found reduced lysozyme of Paneth cell and attenuated autophagic response in VDR knockout mice, and the mice presented a high sensitivity to indomethacin-induced intestinal mucosal injury (Lu et al., 2021). Targeting VDR may inhibit intestinal inflammation and establish host defense against intestinal bacteria, and treatment of NSAID-associated enteropathy with VDR activators can be explored in the future.

Deoxycholic acid (DCA) enhanced ileal lymphocyte migration in part by up-regulating adhesion molecule expression through sphingosine-1-phosphate receptor 2 (S1PR2), and S1PR2 antagonist alleviated indomethacin-induced and DCA-exaggerated small bowel injury (Shibuya et al., 2021). Bile acid receptor S1PR2 may play a proinflammatory role in the intestine.

### 3.4 Microbe–Host Interactions

#### 3.4.1 Bacteria and Bacterial Components

The NSAID-induced small intestinal injury was associated with bacterial overgrowth (Dalby et al., 2006; Kim et al., 2005; Mayo et al., 2016; Muraki et al., 2014; Reuter et al., 1997). NSAID-induced dysbiosis is associated with an expansion of gram-negative bacteria. There is a high positive correlation between the overgrowth of several gram-negative microbial families and the degree of intestinal mucosal injury, while some gram-positive microbial families correlated negatively with the severity of damage (Blackler et al., 2015; Lazar et al., 2021). During inflammation, dysbiosis could be observed in the ileum in rats, as manifested by changes in the relative composition of the microbiota. The biodiversity was not changed (Teran-Ventura et al., 2014). However, Indomethacin-induced enteritis was associated with a reduction in bacterial diversity in the analysis of intestinal bacteria in mice (Imaeda et al., 2012). The difference between the two experiments regarding biological abundance may be due to different animal strains and housing conditions.

Mice with different genotypes had different intestinal microbial compositions. After the administration of the same NSAID, different degrees of small intestinal injury could be seen (Park et al., 2020). Besides, no ulcers were observed in germ-free rats treated with NSAIDs (Robert and Asano, 1977; Uejima et al., 1996), which indicates bacterial flora may play an important role in developing NSAID-associated enteropathy. The incidence of bacterial adherence increased during inflammation, and almost all the bacterial groups were found adhering to the ileal wall (Porras et al., 2004; Teran-Ventura et al., 2014), perhaps this explains the higher lesion index in the distal small bowel than in the proximal part (Satoh and Urushidani, 2016). Bacterial colonization increases the susceptibility to intestinal damage caused by NSAIDs. *E. coli* has been reported to aggravate NSAID enteropathy. After the heated *E. coli* strain administration, the number of ileal ulcers was significantly increased (Hagiwara et al., 2004). Gnotobiotic rats (either administered *Eubacterium limosum* strain ATCC8480 or *Escherichia coli* strain W3110) had a higher ulcer formation rate than germ-free rats after BFMeT administration. However, it did not induce ulcers in any gnotobiotic rats treated with antibiotics (Uejima et al., 1996).

The application of antibiotics may be an effective preventive measure. Treatment with the bactericidal antibiotic polymyxin B, ampicillin, ciprofloxacin significantly inhibited NSAID-induced ulcers (Konaka et al., 1999; Park et al., 2020; Zhong et al., 2016). We can also use probiotics to improve intestinal inflammation. It has been verified in both humans and animals. In a clinical trial, a probiotic mixture containing *Lactobacilli*, *Bifidobacteria*, and *Streptococcus salivaris* significantly reduced fecal calprotectin concentrations in subjects and effectively reduced NSAID-induced intestinal inflammation (Montalto et al., 2010). Bifidobacterium breve Bif195 can safely reduce the risk of acetylsalicylic acid-induced small intestinal mucosal injury without affecting the cardiovascular protective properties of acetylsalicylic acid (Mortensen et al., 2019). Lactobacillus Plantarum exhibited regulation of mucosal structural remodeling on gene transcription level. It demonstrated relatively remarkable probiotic properties (Mujagic et al., 2017). One-week pretreatment with viable *Lactobacillus casei* strain Shirota increased lactic acid concentration in small intestinal contents, which exerted a preventive effect on indomethacin-induced enteropathy (Watanabe et al., 2009). Supplementation with probiotics (*Lactobacillus casei* and *Lactobacillus paracasei*) inhibited oxidative stress and inflammatory factor expression, corrected defective antimicrobial activity, enhanced the intestinal epithelial barrier, ensuring intestinal health in patients treated with NSAIDs (Monteros et al., 2021). The combination of *Bifidobacterium longum* and *lactoferrin* may play a protective role against diclofenac-induced small intestinal injury by inhibiting inflammation through modulation of the TLRs/NF-κB pathway (Fornai et al., 2020a). Changes in the gut microbiota affect the body’s susceptibility to NSAID-induced intestinal injury, and the transplantation of beneficial microbiota can improve this susceptibility. It is believed that it is also a therapeutic hotspot for enteropathy in the future.

Essentially, endotoxin derived from gram-negative bacteria may play a key role in NSAID-associated enteropathy. The primary chemical constituents of bacterial endotoxins are lipopolysaccharides (LPS). Intestinal inflammation triggered by activation of the LPS/Toll-like receptor signaling pathway is a crucial mechanism (Watanabe et al., 2009). Rats treated with LPS developed more scattered ulcers than LPS-untreated rats. LPS increased the number of scattered ulcers in the small intestine dose-dependent (Koga et al., 1999). Add LPS after administration of indomethacin or diclofenac aggravated small intestinal mucosal injury (Watanabe et al., 2008b; Xu et al., 2021).

#### 3.4.2 Toll-like Receptor 4

The innate immune sensors PRRs-toll-like receptors (TLRs) can trigger the innate immune system through various stimuli, such as exogenous microorganisms or endogenous danger signals (Mathur et al., 2018). Activation of the TLR4 signaling pathway could be mediated by LPS or HMGB1 (Boelsterli et al., 2013; Buchholz and Bauer, 2010; Nadatani et al., 2012; Poltorak et al., 1998). TLR4 stimulated downstream signaling events such as nuclear factor-κB (NF-κB) and mitogen-activated protein kinase pathways to induce tissue damage (Kawai and Akira, 2006).

Researchers have suspected that the occurrence of NSAID-associated enteropathy is related to Toll-like receptors. The expression of TLR-4 was upregulated in the small intestine in NSAIDs-treated rats (Teran-Ventura et al., 2014; Xu et al., 2021). The upregulation of TLRs may result from bacteria-host interactions (Tanaka, 2008). The regulation of TLRs relies on an intelligent bacterial recognition system. TLRs are upregulated when the intestinal flora is out of balance, whereas TLRs are relatively down-regulated under normal conditions to reduce host-bacterial interactions (Teran-Ventura et al., 2014). Indomethacin-induced small intestinal mucosal injury and inflammatory cytokine expression were markedly suppressed in TLR4 mutant mice, and indomethacin may damage the small intestine *via* a TLR4/MyD88-dependent pathway (Watanabe et al., 2008a). Appropriate activation of TLRs signaling pathways is beneficial to the body, but their uncontrolled sustained activation is of importance in inducing chronic inflammation. TLR2 agonists lipoarabinomannan (LAM) may attenuate indomethacin-induced small intestinal mucosal lesions and leukocyte infiltration by inhibiting the TLR4 signaling pathway in tissue macrophages (Narimatsu et al., 2015). Rebamipide ameliorates NSAID-induced enteropathy by inhibiting the TLR4/NF-κB signaling pathway (Xu et al., 2021). Bifidobacterium longum, in combination with lactoferrin, may prevent the deleterious effects of NSAIDs by modulating the TLR-2/-4/NF-κB signaling pathway (Fornai et al., 2020b).

### 3.5 Innate Immune System and Inflammatory Response

#### 3.5.1 Innate Immune Cells

Monocytes are involved in the development of inflammation. Yamada et al. demonstrated that monocytes produced IL-17A in indomethacin-induced small bowel inflammation in mice. He used IL-17A (−/−) mice to reveal an essential role for IL-17A in the development of indomethacin-induced small intestinal injury. IL-17A played a proinflammatory role through the up-regulation of G-CSF, KC, and MCP-1, all of which are chemokines. IL-17A may be a new target for treating NSAID-associated enteropathy (Yamada et al., 2011). As this experiment was only performed with gene knock-down mice, immunoneutralization by treatment with anti-IL-17A antibody is needed in future studies.

Macrophages may also be involved in the regulation of non-steroidal anti-inflammatory drug-related enteropathy. Following the administration of indomethacin, researchers found that F4/80+ macrophages were increased in the ulcerated lesions. Clodronate functionally inhibited macrophages, thereby alleviating small intestinal mucosal injury in indomethacin-administered mice (Park et al., 2020). HO-1 exerted a protective effect against NSAID-induced enteritis, HO-1-immunopositive cells accumulated in the small intestine were confirmed to be mainly F4/80 positive macrophages (Harusato et al., 2011). HO-1 is a microsomal rate-limiting enzyme, which has anti-inflammatory and cytoprotective roles. HO-1 protects against oxidative damage and significantly reduces leukocyte chemotaxis and inflammatory factor production (Alcaraz et al., 2003). High expression of HO-1 could protect the intestinal mucosa from indomethacin-induced injury in BACH1-deficient mice (Harusato et al., 2011). HO-1 could be induced *via* the administration of hemin (Yoriki et al., 2013), melatonin (Wu et al., 2012), or camellia oil (Cheng et al., 2014). The translocation of HO-1 from the ER to mitochondria is a cytoprotective mechanism that counteracts NSAID-induced gastrointestinal mucosal injury (Bindu et al., 2015). It appears that macrophages play both pro-inflammatory and anti-inflammatory roles in the intestine. It is not unusual for macrophages to show a dual role in inflammation. A study on alveolar echinococcosis revealed a dual role for hepatic macrophages, which researchers attributed to the differentiation of macrophages into different cell phenotypes at different stages (Wang et al., 2020).

Activation of NF-κB and MAPK in monocytes/macrophages may play a key role in NSAID-induced enteritis by inducing inflammatory cytokines (Nadatani et al., 2012). NF-κBp65 was significantly upregulated in the lamina propria of small intestinal mucosa in NSAIDs-treated rats (Xu et al., 2021). A quinazoline-based BET inhibitor improved indomethacin-induced enteropathy by inhibiting the expression of inflammatory cytokines *via* attenuation of NF-κB and MAPK pathways (Noguchi et al., 2021).

Neutrophil-mediated inflammation has been implicated in NSAID-induced small bowel injury (Stadnyk et al., 2002). Ly6G + neutrophils were detected in the ulcers of indomethacin-treated mice rather than the control WT mice (Park et al., 2020). MPO activity represents the degree of neutrophil infiltration in the intestinal mucosa. There was a positive correlation between the small intestinal tissue injury index and MPO activity (Koga et al., 1999). The MPO activity was markedly elevated in response to indomethacin (Kunikata et al., 2002; Yamada et al., 2011). The elevation in MPO activity was associated with COX inhibition, bacteria, inducible nitric oxide synthase activity, and intestinal hypermotility (Evans and Whittle, 2003; Hatazawa et al., 2006; Satoh et al., 2009). Indomethacin treatment resulted in a 40-fold increase in MPO levels in the ileum compared with control values, much higher than that in the cecum–colon tissues (Teran-Ventura et al., 2014). It can be speculated that indomethacin is more suitable for creating small intestinal inflammation models than for creating acute colitis models. Stadnyk et al. found that CD11a/CD18 and CD11b/CD18 act as leukocyte-specific membrane receptors that mediate neutrophil migration to sites of indomethacin-induced small intestinal injury, neutralizing antibodies against CD11a or CD11b reduce neutrophil infiltration (Stadnyk et al., 2002). Expression of CD11b/CD18 on leukocytes was enhanced in indomethacin-induced rats, and the use of neutralizing antibodies against CD11b could attenuate intestinal injury (Krieglstein et al., 2001).

Epithelial barrier damage and the initial redistribution of CD103 + DC could be seen in the early stages of indomethacin-induced enteritis. The distribution of CD103 + DC could be regulated *via* TLR-2 and -4 antagonism (Silva et al., 2008).

By the way, the adaptive immune system does not seem to play an essential role in NSAID-associated enteropathy. Mice lacking mature T and B cells showed no significant difference in small intestinal damage caused by indomethacin compared with wild-type mice, revealing that T and B cells are not essential for NSAID-induced intestinal injury (Beck et al., 2000). An experiment involving athymic nude rats and Wistar rats demonstrated that indomethacin-induced enteropathy is independent of the role of T cell immunity but is related to environmental factors (Koga et al., 1999).

#### 3.5.2 Cytokines/Enzymes/Receptors in Inflammation

TNF-α has been shown to play an important role in inducing intestinal injury (Fukumoto et al., 2011). TNF-α contributes to neutrophil chemotaxis and activates NF-κB, thereby upregulating the expression of various genes involved in inflammatory responses (Papadakis and Targan, 2000; Stallmach et al., 2004). The TNFα levels in serum or the supernatant of small intestinal mucosal homogenates were significantly increased in NSAID-induced enteritis compared to the control group (Harusato et al., 2011; Tawfik et al., 2016; Xu et al., 2021). Indomethacin induced small intestinal injury in both rats and mice, with increased expression of both TNF-α and MCP-1 (Watanabe et al., 2008b). TNF-α production was directly proportional to indomethacin dosage and was closely related to ulcer area, and inhibition of TNF-α synthesis can significantly reduce small intestinal ulcers (Bertrand et al., 1998). Anti-TNF therapy could protect patients who have been taking long-term NSAIDs from small bowel injury (Watanabe et al., 2014).

The expression of IL-1β was significantly increased after diclofenac administration (Xu et al., 2021). NLRP3 inflammasome-derived IL-1β played a crucial role in NSAID-induced enteropathy. The indomethacin-induced damage in the small intestine was attenuated by blocking IL-1β. However, it was aggravated by using exogenous IL-1β (Higashimori et al., 2016). IL-1β induces the production of other inflammatory factors in the gut (Dinarello and Wolff, 1993). Resolvin D1, a pro-resolving lipid mediator, could ameliorate NSAID-associated enteropathy by inhibiting the expression of TNF-α and IL-1β *via* inhibition of related signaling pathways (Kuzumoto et al., 2021).

Inducible nitric oxide synthase (iNOS) could be induced by cytokines and bacterial lipopolysaccharide to produce sustained and large amounts of NO(Whittle, 1997). NO in the inflammatory environment may produce many cytotoxic fractions in which reactive oxygen species are produced by both inflammatory cells and related tissue. Subcutaneous injection of indomethacin significantly increased iNOS activity in the intestinal mucosa (Kunikata et al., 2002). iNOS can be used as one of the markers of intestinal injury.

Lysozyme, an antimicrobial protein produced by Paneth cells, is involved in the innate immune response. It promotes the lysis of bacteria, and it effectively protects against inflammation at mucosal sites (Ragland and Criss, 2017). Researchers have found that lysozyme expression was lower in indomethacin-treated rats compared to the control group (Bessette et al., 2016).

In addition, appropriate innate immune responses to microbes require complex interactions of several receptors, such as TLRs, protease-activated receptors (PARs) (Chung et al., 2010). We have mentioned the role of TLRs in NSAID-related enteropathy above, and the role of PARs also deserves our attention. PARs are considered important regulators of intestinal homeostasis. PAR2 seemed to play an essential role in the pathogenesis of NSAID enteropathy, while PAR1 seemed to exert a protective effect. PAR1 activation and PAR2 inhibition could be used as a suitable means to prevent NSAID enteropathy (Fornai et al., 2020a). Teprenone may protect the small bowel against diclofenac-induced damage by inhibiting the expression of PAR1 and PAR2(Chao et al., 2021).

### 3.6 Food Intake

NSAID-associated enteritis is associated with feeding conditions. No lesions were found in the small intestine of fasted rats. The intestinal injury was observed in some rats fed continuously, and intestinal lesions were detected in most rats refed after starvation in the predrug period (Weissenborn et al., 1985). Food antigens act synergistically with non-steroidal anti-inflammatory drugs to exacerbate intestinal inflammation (Ma et al., 2021).

Food intake has been demonstrated to be important for the development of intestinal lesions. Sustained reduction of small intestinal lamina propria lymphocytes after a high-fat diet feeding exacerbated indomethacin-induced small intestinal injury (Tanaka et al., 2020). Insoluble dietary fiber plays a vital role in NSAID-induced intestinal lesions (Satoh, 2010). On the other hand, soluble dietary fiber such as pectin, guar gum, and sodium alginate could significantly reduce lesion formation and prevent NSAID-induced small intestinal injury in rodents (Horibe et al., 2016; Satoh et al., 2016; Satoh and Urushidani, 2016). In recent years, researchers have taken a new step in dietary therapy. Preoral administration of camellia oil effectively reduced ketoprofen-induced oxidative gastrointestinal injury through upregulation of vascular endothelial growth factor (VEGF) (Cheng et al., 2014), and this study broadened the beneficial effects of VEGF from gastric mucosal protection to intestinal mucosal protection. Taking bovine colostrum was beneficial in preventing small intestinal injury caused by NSAIDs (Kim et al., 2005). An immune-modulating diet containing whey peptides and fermented milk products exerted a protective effect on the indomethacin-treated small intestine (Kume et al., 2014). Milk fermented with Lactobacillus fermentum may effectively reduce intestinal inflammation in mice after the administration of NSAIDs (Santiago-Lopez et al., 2019). A randomized controlled trial revealed that yogurt containing Lactobacillus gasseri reduced gastrointestinal symptoms caused by aspirin (Suzuki et al., 2017).

### 3.7 Autophagy Inhibition

NSAID-induced autophagy inhibition impaired the integrity of the mucus layer combined with the inability of intestinal epithelial cells to clear intracellular pathogens, which elicited an uncontrolled inflammatory response eventually (Chamoun-Emanuelli et al., 2019). Pure total flavonoids from citrus could promote autophagy to protect the small intestinal barrier by activating the PI3K/Akt signaling pathway (Chen et al., 2021). It is a flavonoid isolated and purified from citrus peel, and its medicinal value for gastrointestinal protection has been demonstrated.

### 3.8 Genetic Susceptibility

Genetic susceptibility is strongly associated with intestinal inflammation. In genetically susceptible Lewis rats, the plasma kallikrein-kinin system was activated, associated with both acute and chronic phases of intestinal injury (Stadnicki et al., 1998).

### 3.9 Psychological States

It has been reported that psychological stress and corticotropin-releasing hormone increase intestinal permeability in humans, which is inhibited by mast cell stabilizers, revealing a close association between the central nervous system and gastrointestinal function in humans (Vanuytsel et al., 2014).

In a water avoidance stress model, psychological stress caused changes in intestinal microbiota, increased intestinal permeability, and deterioration of NSAID-associated enteropathy, all of which were ameliorated by the GR antagonist, mifepristone (Yoshikawa et al., 2017).

5-HT is a neurotransmitter. A large amount of 5-HT is localized in the intestine, especially in enterochromaffin cells (Gershon and Tack, 2007). Endogenous 5-HT is involved in the development of indomethacin-induced enteropathy. On the one hand, it exerted a pro-ulcer effect *via* the 5-HT3 receptor. On the other hand, it exerted an anti-ulcer effect *via* the 5-HT4 receptor (Kato et al., 2012).

### 3.10 Drug Interaction

Some researchers found PPI and H2RA as relative risk factors when studying small intestinal injury in people taking non-steroidal anti-inflammatory drugs (Endo et al., 2014; Watanabe et al., 2013). A significant worsening of intestinal damage was observed in animals co-administered with PPIs in either rats given a selective COX-2 inhibitor (celecoxib) or naproxen. Long-term use of PPIs in rodents led to an exacerbation of indomethacin-induced small intestinal damage by inducing dysbiosis (Wallace et al., 2011). The use of proton pump inhibitors is closely related to small intestinal bacterial overgrowth (Lo and Chan, 2013). Besides, the application of PPIs inhibits contractile activity and frequency in the distal part of the small intestine, and the inhibition of intestinal contractility is associated with increased inflammation. The PPI-induced reduction in intestinal contractility appears to be due to increased IFN-γ and NF-κB activity (Lichtenberger et al., 2015).

However, lansoprazole prevented the development of indomethacin-induced intestinal damage in a dose-dependent manner (Kuroda et al., 2006). In indomethacin-induced intestinal models, treatment with H2RA had an inhibitory effect on the inflammation of intestinal mucosal tissues, and it increased the defense function of epithelial cells (Kawashima et al., 2020). At present, there is still controversy in the effect of acid-suppressing drugs on NSAID-induced small intestinal injury, and this issue needs to be further explored by subsequent experiments.

Dexamethasone is a commonly used glucocorticoid in clinical practice. Its two targeted nuclear receptors, glucocorticoid receptor (GR) and peroxisome proliferator-activated receptor α(PPARα), may play an essential role in regulating NSAID-related enteropathy. Dexamethasone aggravated ibuprofen-induced intestinal toxicity *via* GR signaling. It also increased the enterohepatic circulation of ibuprofen and its acyl glucuronide by inducing PPARα-glucuronosyltransferase signaling to aggravate the intestinal injury (Lu et al., 2018).

Protease inhibitor therapy in AIDS patients increases their risk of cardiovascular disease, and they routinely use aspirin for prophylaxis. However, the protease inhibitor ritonavir increased aspirin-induced intestinal mucosal injury without increasing gastric mucosal injury (Renga et al., 2014).

## 4 Conclusion

In summary, Indomethacin and diclofenac are more widely used drugs in the current NSAID-induced small intestinal injury model. NSAIDs’ systemic and local effects trigger the development and persistence of small intestinal inflammation in rodents. NSAID-related enteropathy results from multiple factors that complement each other, we have summarized the pathogenetic factors in Table 2 and protective factors in Table 3. Future studies should focus on exploring the causal relationship between various etiological factors. However, enteritis involved in numerous experiments is a simple model of acute NSAID-associated injury rather than chronic inflammation. Some investigators erroneously interpret the NSAID-induced acute enteritis model as a model of human NSAID-associated enteropathy simply due to the villous destruction and inflammatory cell infiltration that occurs in the acute phase. NSAID-associated enteropathy is intestinal damage caused by long-term medication. Prolonged modeling time in animals may better reflect the results induced by NSAIDs in humans. New therapeutic strategies can be effectively developed only by using appropriate animal models.

**TABLE 2:** Pathogenesis of NSAID-associated small intestinal injury.

Categories	Factors (References)
Cells	F4/80+ macrophage Park et al. (2020), Monocyte Yamada et al. (2011), Ly6G + neutrophil Park et al. (2020), CD103 + DC Silva et al. (2008)
Cytokines/chemokines	TNF-α Fukumoto et al. (2011); Bertrand et al. (1998); Watanabe et al. (2014), IL-1β Higashimori et al. (2016), IL-17A Yamada et al. (2011)
Receptors	PAR2 Fornai et al. (2020b); CD11a/CD18 Stadnyk et al. (2002), CD11b/CD18 Stadnyk et al. (2002); Krieglstein et al. (2001), 5-HT3R Kato et al. (2012), GRLu et al., (2018); Yoshikawa et al. (2017), PPARα (Lu et al. (2018) S1PR2 Shibuya et al. (2021)
Transcription factors	NF-κB p65 Xu et al. (2021); Nadatani et al. (2012), Noguchi et al. (2021), MAPK p38 Nadatani et al. (2012), Noguchi et al. (2021)
Toll-like receptors	TLR4Watanabe et al. (2008a), Narimatsu et al. (2015), Xu et al. (2021)
Enzymes	COX inhibition Walker et al. (2014); Somasundaram et al. (2000), Beck et al. (1990), iNOS Kunikata et al. (2002), Whittle (1997)
Bacteria and bacterial components	Eubacterium limosum Uejima et al. (1996), *Escherichia coli* Hagiwara et al. (2004), Uejima et al. (1996), LPS Watanabe et al. (2009), Koga et al. (1999)
Enterohepatic circulation	Bile Robert and Asano, (1977), Yamada et al., (1993), Zhou et al. (2010), Bacterial β-d-glucuronidase (LoGuidice et al. (2012), Saitta et al. (2014), Zhong et al. (2016)
Food intake	Food antigen Ma et al. (2021), High-fat diet Tanaka et al. (2020), Insoluble dietary fiber Satoh (2010)
Genetic susceptibility	K-K system activation Stadnicki et al. (1998)
Oxidative stress	Uncoupling of oxidative phosphorylation Somasundaram et al. (1997), Somasundaram et al. (2000), ROS Bhatt et al. (2018), Omatsu et al. (2009)
Autophagy	Autophagy inhibition Chamoun-Emanuelli et al. (2019)
Drug interactions	PPI Endo et al. (2014), Watanabe et al. (2013), H2RA Watanabe et al. (2013) Dexamethasone Lu et al. (2018) Ritonavir Renga et al. (2014)

**TABLE 3:** Prevention of NSAID-associated small intestinal injury.

Categories	Factors (References)
Cells	F4/80+ macrophage Harusato et al. (2011)
Growth factors	VEGF Cheng et al. (2014)
Receptors	PAR1 Fornai et al. (2020a), 5-HT4R Kato et al. (2012), FXR Fiorucci et al. (2011), PXR Venkatesh et al. (2014), TGR5 Cipriani et al. (2013) VDR Lu et al. (2021) EP3R Kunikata et al. (2001), Kunikata et al. (2002), EP4R Kunikata et al. (2001), Kunikata et al. (2002)
Enzymes	Lysozyme Ragland and Criss (2017), Bessette et al. (2016), HO-1 Harusato et al. (2011), Bindu et al. (2015)
Antibiotics	Polymyxin B Park et al. (2020), Ampicillin Konaka et al. (1999), Ciprofloxacin Zhong et al. (2016)
Probiotics	Lactobacilli and Bifidobacteria and Streptococcus salivaris Montalto et al. (2010), Bifidobacterium breve Mortensen et al. (2019) Lactobacillus plantarum Mujagic et al. (2017) Lactobacillus casei strain Shirota Watanabe et al. (2009) Lactobacillus casei and Lactobacillus paracasei Monteros et al. (2021) Bifidobacterium longum and lactoferrin Fornai et al. (2020b)
Enterohepatic circulation	Bile duct ligation Brodie et al. (1970), Somasundaram et al. (1997)
Chemically modified NSAIDs	NO-NSAID Reuter et al. (1997), H2S-NSAID Wallace et al. (2015), Wallace et al. (2010), PC-NSAID Dial et al. (2015), Lanza et al. (2008), NSAID-antioxidant conjugates Ashraf et al. (2016), Amino-alcohol ester derivatives Halen et al. (2006)
Dietary therapy	Soluble dietary fiber Horibe et al. (2016); Satoh et al. (2016), Satoh and Urushidani (2016), Camellia oil (Cheng et al. (2014), Bovine colostrum Kim et al. (2005), Whey peptides and fermented milk Kume et al. (2014), Milk fermented with Lactobacillus fermentum Santiago-Lopez et al. (2019) Yogurt containing Lactobacillus gasseri Suzuki et al. (2017)
Acid-suppressing drugs	PPI Kuroda et al. (2006), H2RA Kawashima et al. (2020)
Antioxidant	Polaprezinc Watari et al. (2013)
Natural products	Pure citrus total flavonoids Chen et al. (2021), Catechin Cheng et al. (2013), Essential oil of Citrus limon Bouzenna et al. (2019), Epiisopiloturine hydrochloride Nicolau et al. (2017), Cashew gum Carvalho et al., 2015)
Hormones	Melatonin Sanchez et al. (2020)

Regarding the construction of disease models, most experiments use *in vivo* animal models or *in vitro* human cell line models. The latest study reports that enterocyte-like cells derived from human embryonic stem cells are consistent with human cell line trends in toxicological reactions. They have a better correlation with *in vivo* models (Ryu et al., 2021). They may be used as an animal model alternative to solve the ethical problems involved in future studies.

Many older people have common underlying diseases such as hypertension and diabetes. These patients need to take a certain number of drugs every day. The liver is the principal organ of drug metabolism. Efforts should be made to avoid the use of other drugs that increase liver drug metabolism to improve NSAID-associated intestinal injury. Therefore, the application of dietary therapy and chemically modified NSAID derivatives are promising.

Many natural products have anti-inflammatory and antioxidant properties, and they have had some success in treating NSAID-related enteropathy. Besides, natural products have fewer side effects, low cost, and high availability, so the development of such drugs is also promising in the future.

Future studies can focus on drug interactions for NSAID-induced intestinal injury and establish guidelines to guide clinical prescription to circumvent the risk of increased harm following drug interactions.
